# Size Effects of Au/Ni-Coated Polymer Particles on the Electrical Performance of Anisotropic Conductive Adhesive Films under Flexible Mechanical Conditions

**DOI:** 10.3390/ma17071658

**Published:** 2024-04-04

**Authors:** Yexing Fang, Taiyu Wang, Yue Gu, Mingkun Yang, Hong Li, Sujun Shi, Xiuchen Zhao, Yongjun Huo

**Affiliations:** 1 School of Materials Science and Engineering, Beijing Institute of Technology, Beijing 100081, China; 2 Department of Neurosurgery, Yale University, New Haven, CT 06520, USA

**Keywords:** anisotropic conductive adhesive films, thermo-compression bonding, flexible mechanics, electrical properties, conductive particle, fine-pitch interconnection

## Abstract

In soft electronics, anisotropic conductive adhesive films (ACFs) are the trending interconnecting approach due to their substantial softness and superior bondability to flexible substrates. However, low bonding pressure (≤1 MPa) and fine-pitch interconnections of ACFs become challenging while being extended in advanced device developments such as wafer-level packaging and three-dimensional multi-layer integrated circuit board assembly. To overcome these difficulties, we studied two types of ACFs with distinct conductive filler sizes (ACF-1: ~20 μm and ACF-2: ~5 μm). We demonstrated a low-pressure thermo-compression bonding technique and investigated the size effect of conductive particles on ACF’s mechanical properties in a customized testing device, which consists of flexible printing circuits and Flex on Flex assemblies. A consistency of low interconnection resistance (<1 Ω) after mechanical stress (cycling bending test up to 600 cycles) verifies the assembly’s outstanding electrical reliability and mechanical stability and thus validates the great effectiveness of the ACF bonding technique. Additionally, in numerical studies using the finite element method, we developed a generic model to disclose the size effect of Au/Ni-coated polymer fillers in ACF on device reliability under mechanical stress. For the first time, we confirmed that ACFs with smaller filler particles are more prone to coating fracture, leading to deteriorated electrical interconnections, and are more likely to peel off from substrate electrode pads resulting in electrical faults. This study provides guides for ACF design and manufacturing and would facilitate the advancement of soft wearable electronic devices.

## 1. Introduction

In the past decade, there has been significant progress in flexible electronics manufacturing technology and widespread adoption of wearable devices. This has led to a continuous reduction in the size of flexible electronic devices such as flexible displays and flexible circuit boards [1,2,3]. To address the demands of this trend, the flex-on-flex (FOF) assembly structure was pursued owing to its numerous advantages, including flexibility, space efficiency, miniaturization, and fine-pitch designs [4]. However, as electronic devices continue to shrink in size, the unique characteristics of flexible materials, such as their flexibility and extensibility, have posed challenges in component manufacturing. Consequently, it becomes imperative to engineer adaptable electronic package structures and interconnect materials [5,6,7,8]. In the realm of traditional microelectronic packaging, flip chip technology with a face-array structure has gained popularity because of its outstanding features, including quick heat dissipation, high I/O density, and robust electrical, thermal, and mechanical connections. It has become one of the leading technologies for advanced integrated circuit chip packaging [9,10,11,12,13]. The advantages and characteristics of flip-chip technology also extend to flexible electronic packaging. By utilizing flexible materials, products are more malleable and bendable, as well as showing size reduction and improved space usage [14]. Consequently, flip-chip technology has emerged as one of the most favorable packaging techniques in the flexible electronics field [15,16,17]. In addition, the electronics industry is actively embracing environmentally friendly interconnect materials for green process manufacturing and green packaging, thus complying with the concept of “green production.” Anisotropic conductive film (ACF) is a widely used electronic component, consisting of conductive particles and adhesive polymer resins [18,19,20,21]. By subjecting the ACF to specific temperature and pressure conditions [22], the conductive particles within the ACF are compressed vertically (in the *Z*-axis direction), facilitating an electrical conduction pathway [23]. Simultaneously, there is no in-plane contact between these conductive particles (*X*-axis and *Y*-axis directions), ensuring insulation [24]. ACF enables flexible electronic devices to function normally under bending and stretching, thanks to its strong adhesion force and reliable electrical conductivity [25,26]. Due to its fine-pitch capability, green material properties, and straightforward process, ACF has found applications in various areas, including laptop displays, flexible printed circuit boards (FPCBs), chip-on-chip, chip-on-glass, FOF, and flip-chip semiconductor packaging [27,28,29]. Overall, ACF demonstrates promising prospects for flexible electronics packaging [30].

In the application of wafer-level packaging (WLP), which requires low-pressure bonding and fine-pitch interconnections, the primary challenge faced by ACF is the electrical short-circuit between neighboring electrodes [31,32,33]. The size of conductive particles has been identified as the primary cause of this problem [34]. In recent years, researchers have explored the use of ACFs in fine-pitch flexible assemblies. Unlike electronic materials employed in traditional rigid substrates, flexible electronic materials utilized in wearable electronics encounter challenges related to flexible mechanics [35], such as bending, stretching [36], and fatigue mechanics induced by cyclic small forces [37]. Currently, most studies have focused on examining the mechanical and electrical properties of ACFs made from different materials in flexible substrates. As an example, Ji-Soo Lee et al. achieved more stable fine-pitch FOF assemblies by incorporating an optimized post-flux activator into nanofiber solder ACF [38]. Shuyue Zhang and Sun-Chul Kim et al. [39,40,41] investigated various applications of ACFs in 100 μm fine-pitch flexible printed circuits (FPCs) and achieved improved electrical properties and enhanced reliability of FOF by optimizing the material properties of ACF components. However, there is a lack of analysis concerning the size effects of conductive particles on the performance of ACFs under flexible mechanical conditions.

**Figure 1:**
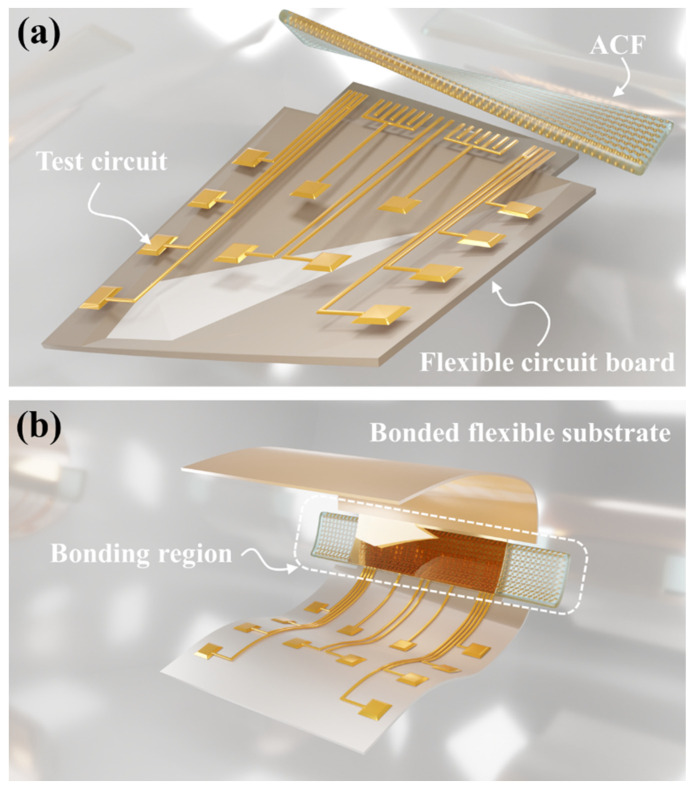
(**a**) The image of the polyimide based flexible printed circuits (FPCs) and the anisotropic conductive adhesive film (ACF); (**b**) The schematic diagram of Bonded FOF with ACF in flexible application.

In this study, two commercially available ACFs were utilized as interconnection materials in Flex on Flex (FOF) assemblies, namely ACF-1 (particle diameter ~20 μm) and ACF-2 (particle diameter ~5 μm). Both ACFs consist of Au/Ni-coated polymer spheres and adhesive epoxy resins. Firstly, we used the low-pressure thermo-compression bonding technique and a self-built rapid electrical test platform to fabricate nine FOF assemblies with different bonding parameters and evaluate their electrical performance. The electrical performance results indicate that the optimal electrical performance for ACF-1 is achieved at 170 °C and 0.67 MPa, and for ACF-2 at 220 °C and 0.67 MPa. Furthermore, flexible bending tests as well as bending simulations were conducted to compare the electrical performance of the two types of ACFs and analyze the size effects of ACFs during bending processes. Figure 1a illustrates the schematic diagram of the FPCs and ACF used in this study. Figure 1b presents the schematic diagram of a FOF assembly applied in flexible applications.

## 2. Material and Methods

### 2.1. Materials

**Figure 2:**
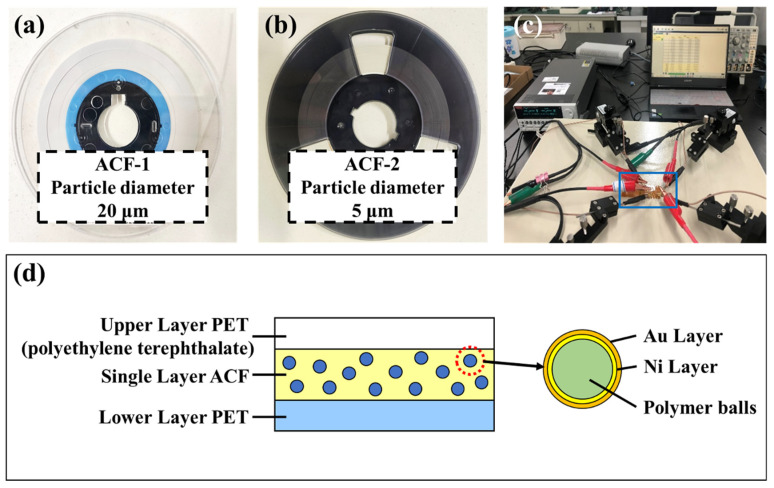
(**a**,**b**) Two types of ACFs, namely ACF-1 (particle diameter ~20 μm) and ACF-2 (particle diameter ~5 μm). (**c**) The self-built rapid electrical test platform. (**d**) The cross-sectional diagram of ACF. The surfaces of ACF are covered with PET (polyethylene terephthalate) protective films. The ACF consists of Au/Ni-coated polymer spheres and adhesive epoxy resins.

In this study, two types of single-layer ACFs, namely ACF-1 from Shenzhen Fisher New Materials Co., Ltd. (Shenzhen, China) and ACF-2 from Hitachi Chemical Co., Ltd. (Tokyo, Japan), were specifically chosen for analysis as depicted in Figure 2a,b. The conductive particle diameter in ACF-1 is ~20 μm, and that in ACF-2 is ~5 μm. The resin matrix of both ACFs is made of epoxy resin, and the conductive particles in both ACFs are made of polymer spheres coated with Ni/Au layers, as illustrated in Figure 2d.

**Figure 3:**
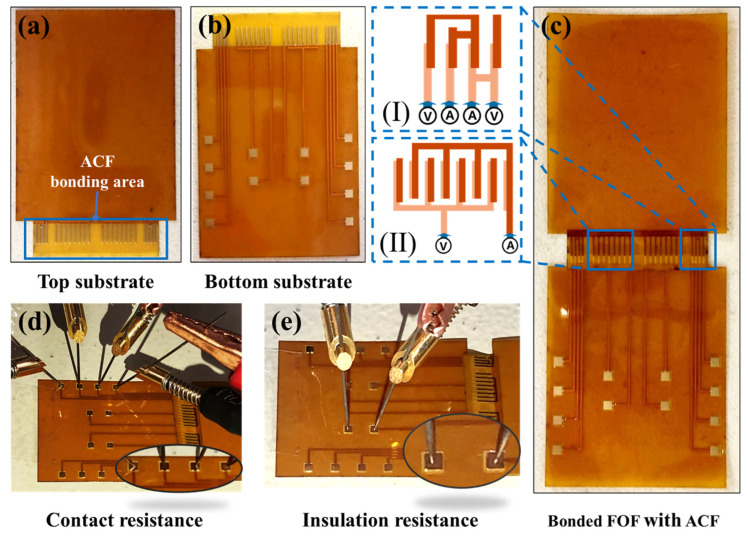
The schematic diagram of experimental materials and electrical test configuration. (**a**,**b**) The top and bottom FPC substrate: the blue box delineates the bonding region where ACF is positioned; (**c**) The bonded Flex on Flex (FOF) sample with ACF, (I) the interconnection structure of contact resistance circuit, (II) the interconnection structure of insulation resistance circuit; (**d**) Contact resistance test configuration; (**e**) Insulation resistance test configuration.

The FPC comprises a top FPC and a bottom FPC substrate, as depicted in Figure 3a,b. The bottom FPC is equipped with four copper pads for measuring contact resistance and two copper pads for measuring insulation resistance, as shown in Figure 3d,e. The FPC is constructed from polyimide (PI), with a thickness of 0.05 mm, a width of 24 mm, and a length of 30 mm. The copper pads are 1.2 mm × 1.2 mm. The bonding region of the FPC has a thickness of 0.05 mm, a width of 18 mm, and a height of 5 mm. Also, the width of the copper trace in the bonding region is 0.25 mm, and the pitch between the copper traces is 0.30 mm.

### 2.2. Bonding Process

The interconnection performance of ACFs is primarily influenced by bonding temperature, time and pressure. The appropriate temperature and time ensure that the adhesive attains a certain degree of curing by providing sufficient heat. Pressure, on the other hand, ensures that the conductive particles in the *Z*-axis direction are deformed by pressure to make contact with each other over a certain area in order to achieve interconnection.

In this study, the low-pressure thermo-compression bonding process was carried out using the flip-chip bonder Finetech PICO 145 (Finetech GmbH & Co. KG, Berlin, Germany). The bonding process was divided into two stages, namely the temporary bonding and the final bonding. The temporary bonding was conducted at a temperature below the resin curing temperature (≤80 °C), which allowed the ACF to be fixed to the bottom FPC substrate before the gel was cured. On the other hand, the final bonding was performed above the resin curing temperature, which allowed the resin to attain a certain degree of cure. Table 1 presents the standard bonding parameters of the two ACFs. In order to identify the bonding parameters that yield optimal electrical performance for the samples under low-pressure bonding conditions, three sets of bonding temperatures and three sets of bonding pressures were established. These parameters were based on the standard final bonding parameters of ACF-1 and ACF-2, both of which have the same gradient of variation. The bonding time used in this study remained consistent with the standard final bonding time. The bonding pressure and temperature for the two ACFs are presented in Table 2. In details, the bonding pressure was fixed at 0.2 MPa, 0.33 MPa and 0.67 MPa, while the bonding temperature for ACF-1 was set to 130 °C, 150 °C and 170 °C, respectively. The bonding temperature for ACF-2 was set to 180 °C, 200 °C and 220 °C. Figure 3c illustrates the ACF-bonded FOF assembly.

### 2.3. Characterization

#### 2.3.1. Scanning Electron Microscopy and Energy Dispersive X-ray Spectroscopy

To verify the reliability of the data provided by the companies and to provide a reference for the establishment of finite element simulation models, an investigation was conducted on the microscopic morphology of ACFs. The microscopic morphology of the ACFs was observed using the Cold Field Emission SEM HITACHI Regulus 8230 (Hitachi High-Tech Co., Ltd., Tokyo, Japan), located in the Experimental Center of Advanced Materials (ECAM). The SEM images presented in this paper were captured in secondary electron (SE) mode with an electron beam voltage of 10 kV and using an Everhart-Thornley (ET) detector to capture the surface morphology of the samples.

The chemical compositions of the ACFs were quantitatively examined using SEM/EDX with the Regulus 8230 SEM. To determine the average solute concentration in each area, the EDX spectrum mapping mode was utilized with the ZAF correction method, with an electron beam voltage of 20 kV and a take-off angle of 33.7°. The results of the SEM/EDX spectrum mapping were collected and summarized, including information on the average solute concentration and machine error.

In order to observe the internal morphology of the ACF, a series of experiments were conducted. Two copper sheets were subjected to ultrasonic cleaning with anhydrous ethanol. Subsequently, the copper sheets were bonded together using each type of ACF with the standard bonding parameters, as depicted in Table 1. The temporary bonding condition for ACF-1 was optimized at 1 MPa, 80 °C, and 2 s bonding, while the final bonding condition was set at 0.67 MPa, 150 °C, and 15 s bonding. Similarly, for ACF-2, the standard temporary bonding condition was set at 1 MPa, 80 °C, and 2 s bonding, while the final bonding condition was set at 0.67 MPa, 200 °C, and 10 s bonding.

#### 2.3.2. Electrical Test

In optimal circumstances, ACFs exhibit conductivity solely in the *Z*-axis direction while remaining insulated in the *X*-axis and *Y*-axis directions during operation. Therefore, this study designed two circuits for the purpose of measuring contact resistance and insulation resistance, as depicted in Figure 3c(I,II). The contact resistance of the two ACFs when interconnected is evaluated using the four-point probe method, while the method of applying voltage to measure current is used to calculate insulation resistance. For details, the contact resistance was measured at three different levels: 0.1 mA, 1 mA, and 10 mA, while the insulation resistance was measured at four different voltage levels: 1 V, 2 V, 10 V, and 20 V. The circuits utilized in the aforementioned methods are depicted in Figure 3c.

In order to ensure the controllability of the electrical performance measurement specification and to mitigate the impact of random errors arising from the experimental environment, a novel electrical test platform was developed in this study, as depicted in Figure 2c. The platform consists of a Keithley 2636B (TEKTRONIX, INC., OR, USA)series digital source meter, probe holders, tungsten probes, a coaxial spring fixture, a magnetic desktop, and Kickstart software (Version 2.8.0) on a personal computer. The schematic diagram depicting the contact between the probe and the copper electrode during the measurement of contact resistance and insulation resistance is presented in Figure 3d,e, respectively.

#### 2.3.3. Bending Test

The bending test samples were prepared through low-pressure thermo-compression bonding, utilizing optimal parameters for both ACFs. The bending test was conducted using a FlexTest-TM-L flexible electronic tester (NanoUp Electronics Technology Co, Ltd., Hunan, China) with a deformation speed of 2 s per cycle at a controlled software panel setting, in accordance with GB/T 38001.61-2019 Mechanical Stress Test Method for flexible display parts [42,43]. The bending test was performed at room temperature (23 °C), and a slow bending speed was employed to prevent excessive heat generation during the experiment. The bending radius of curvature was set to 1 mm, given that the ACF interconnection part of the FOF system has a width of 1.5 mm. The number of bending cycles was set to 10, 50, 100, 200, 400, 600, 800, 1000, 1200, 1500, 1700, and 2000, as well as 5000. A video of the bending procedure can be seen in Video S1.

#### 2.3.4. Modeling and Simulation

To investigate the von Mises stress in the conductive particles under cyclic bending mechanics, the finite element method (FEM) simulation was performed using COMSOL MULTIPHYSICS software (Version 6.1) to simulate the bending process of the two ACFs. Considering the symmetry of the bending process in FOF assemblies, a local model was constructed with the bending central axis as the axis of symmetry, as illustrated in Section 3.4 In detail, the bending models contained two different sizes of conductive particles (10 μm and 20 μm in diameter). In order to streamline the simulation model and decrease the quantity of particles, the conductive particles were simplified as polymer spheres, and the diameter of the smaller particles was modified from 5 μm to 10 μm. The simulations utilized the same materials, spherical center spacing of the conducting spheres, and bending conditions as those used in the bending experiments. The properties of the materials utilized in the simulations are presented in Table 3.

To ensure more precise calculations, a tetrahedral cell with adaptive meshing was utilized [44]. The copper was considered to be plastic, while both the resin matrix and polymer spheres were assumed to be elastic materials. To bridge the gap between simulation and experiment, the ACF joint was designed as an isolated structure through physical contact, with the ACF epoxy resins and the polymer balls forming a one-piece structure. Steady-state simulations were conducted to analyze the deformation and stress–strain states during a single bending. Likewise, the ACF with 20 μm particles is designated as ACF-1, and the ACF with 10 μm particles is referred to as ACF-2.

## 3. Results and Discussions

### 3.1. Morphological Characterization

**Figure 4:**
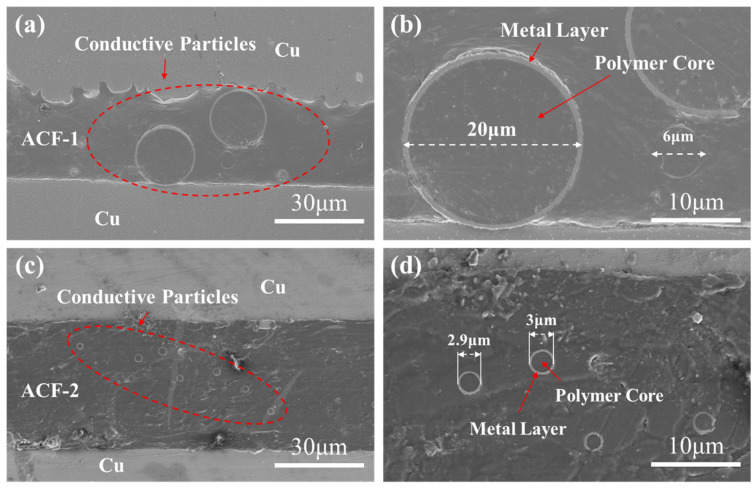
The morphology and distribution of conductive particles of (**a**,**b**) ACF-1 and (**c**,**d**) ACF-2 at different magnifications.

Figure 4 illustrates the morphology of the two types of ACF-bonded cross sections, showcasing the size and distribution density of their conductive particles. As a general rule, each type of ACF is composed of two components: a resin matrix and conductive particles. These conductive particles are polymer microspheres that have been coated with Au/Ni layers. It is noteworthy that the ACF-1 conductive particles exhibit a larger diameter, measuring approximately 20 μm, and a lower particle distribution density per unit area. Meanwhile, the ACF-2 conductive particles possess a smaller diameter, measuring approximately 3 μm, and a higher particle distribution density per unit area. The diameter of the conductive particle in the cross section is less than or equal to the diameter of the conductive particles, due to the dispersed distribution of conductive particles within ACF and the random cross-sectional observation of the sample. It is important to note that the morphology does not necessarily imply that the conductive particles are not in contact with each other, as there may be conductive particles in contact with them in other directions.

**Figure 5:**
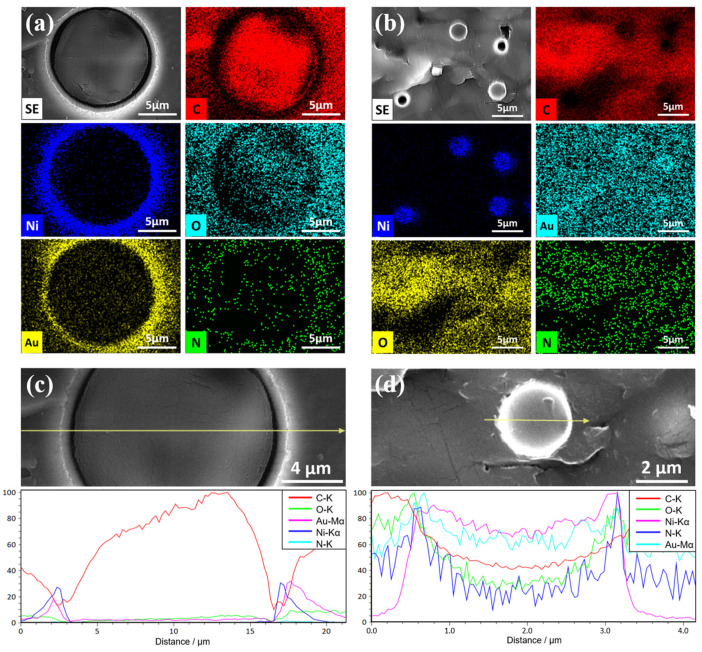
The EDX results of selected elements of carbon, nickel, oxygen, gold and nitrogen. Mapping scan analysis of (**a**) ACF-1 and (**b**) ACF-2, and line scan analysis of (**c**) ACF-1 and (**d**) ACF-2.

The analysis of the two ACFs’ composition and the distribution of conductive particles’ elements can be conducted through EDX. The distribution of the primary elements of ACF-1 and ACF-2 is illustrated in Figure 5. It is evident that the elemental composition and distribution of both conductive particles are analogous. The metallic elements Au and Ni are predominantly distributed in the outer layer of the conductive particles. Conversely, the elements C, O, and N are uniformly distributed in the polymeric core of the conductive particles and the colloidal part of the ACF. The larger conductive particles (ACF-1) exhibit a more distinct distribution of each metal element in comparison to the smaller ones (ACF-2). Additionally, the contours of the spherical polymer core of the conductive particles are also clearly observable. As the ACF-2 sample underwent conductive treatment in SEM, it was sputtered with Au, rendering the visualization of Au element distribution in the outer layer of conductive particles impossible. However, the line scan results depicted in Figure 5d reveal the presence of Au in the outer layer of ACF-2 conductive particles.

After analyzing the SEM and EDX results, it is evident that the outer metal layer elements of ACF-1 conductive particles exhibit distinct distribution characteristics in the surface scan without diffusing to the inner core of the polymer. Conversely, the outer metal layer of ACF-2 conductive particles displays fuzzier distribution characteristics in the surface scan, with prominent diffusion characteristics to the inner core of the polymer.

### 3.2. ACF Bonding Results

**Figure 6:**
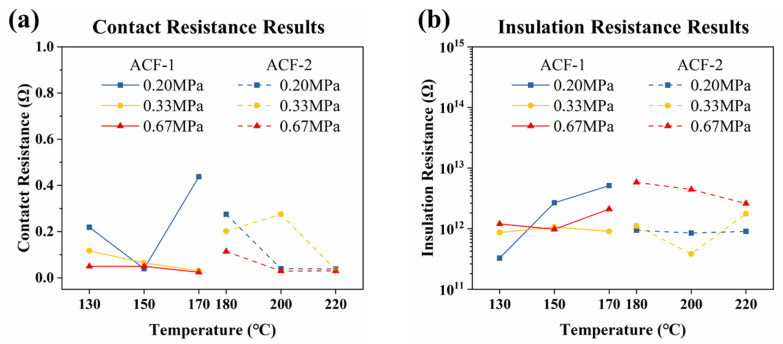
The electrical performance of bonding samples. (**a**) The contact resistance of ACF-1 and ACF-2 measured at 10 mA and (**b**) the insulation resistance of ACF-1 and ACF-2 measured at 20 V.

To ensure the accuracy and reliability of the electrical test results, the authors conducted initial data screening, and the selected test results are presented in Figure 6. The complete electrical test results can be viewed in Figures S1 and S2. Also, the specific screening criteria can be found in the supporting information. Figure 6a illustrates the contact resistance results of ACF-1 and ACF-2 measured at 10 mA, and Figure 6b showcases the insulation resistance results of ACF-1 and ACF-2 measured at 20 V. It can be seen that the bonding pressures are set to 0.20 MPa, 0.33 MPa, and 0.67 MPa for each ACF. Moreover, the bonding temperatures for ACF-1 are set to 130, 150 and 170 °C, while those for ACF-2 are set to 180, 200 and 220 °C. Notably, as depicted in Figure 6a, the contact resistance of both ACFs is observed to be less than 1 Ω, indicating a low ohmic behavior. It suggests that the low-pressure bonding parameters employed in the experiment are capable of achieving a robust electrical connection and further imply that the properties at the ACF interconnection joints are akin to those of metal conductors. As shown in Figure 6b, the insulation resistance of both ACFs is maintained at a level of 10^12^ Ω. It indicates that temperature and pressure have a negligible impact on the horizontal conductivity of particle aggregation, and there is good lateral insulation between the electrodes of the FOF assemblies. This phenomenon can be attributed to the utilization of low-pressure bonding parameters in the experiment. Since the bonding pressures applied in this study are relatively low (the standard bonding pressure is 1 MPa), the deformation of the conductive particles is not significant enough to cause squeezing of the adjacent conductive particles in the horizontal direction. The bonding pressure primarily affects the contact between the conductive particles and the adjacent area in a vertical direction. Therefore, the variation in bonding pressure set in this experiment is unlikely to significantly affect the insulation performance in the horizontal direction.

**Figure 7:**
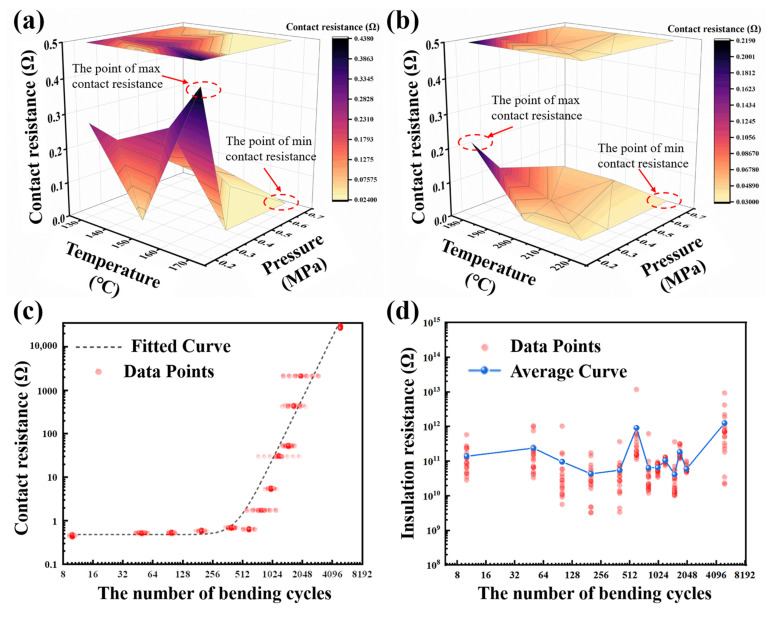
Three-dimensional plot of contact resistance of (**a**) ACF-1 and (**b**) ACF-2 under a 10 mA current. (**c**) Contact resistance of ACF-1 varies with the increasing number of bending cycles. (**d**) Insulation resistance of ACF-1 varies with the increasing number of bending cycles. The bending cycle times are set to 10, 50, 100, 200, 400, 600, 800, 1000, 1200, 1500, 1700, and 2000, as well as 5000.

The electrical performance of ACF joints is typically evaluated through the measurement of contact resistance [45]. In order to provide a more intuitive comparison of the contact resistance values, Figure 7a,b illustrate the 3D plot of the contact resistance of the two ACFs at varying bonding temperatures and pressures, measured at a 10 mA current. The lighter shaded regions correspond to lower contact resistance, indicating superior electrical conductivity. It can be seen that the optimal bonding parameters for the two ACFs to achieve the desired electrical properties are: 170 °C and 0.67 MPa for ACF-1, and 220 °C and 0.67 MPa for ACF-2. It is noteworthy that the bonding temperatures of the two ACFs, as determined in this study, exceed their standard bonding temperatures. This is due to the fact that the bonding pressure utilized in this study is lower than the standard bonding pressure, and a suitable increase in temperature can compensate for the effect of the reduced bonding pressure.

### 3.3. Bending Test Results

Figure 7c illustrates the test results of the contact resistance of ACF-1 as a function of the number of cyclic bending times. To ensure clarity, the electrical data points for each tested sample (20 sets per sample) are rendered transparent, while the points with identical values are expanded horizontally. Figure 7d depicts the trend of the insulation resistance of ACF-1 with increasing cyclic bending times. Given the erratic nature of the data, the average value of the measured data for each substrate is taken as the standard data to showcase the trend of the insulation resistance. As ACF-2 experienced peeling after 200 bending cycles, there are insufficient data to demonstrate the bending results of ACF-2. The bending results of ACF-2 (including only the 10th, 50th, and 100th tests) can be seen in Figure S3 (Supplementary Materials).

In the cyclic bending test, from a mechanical perspective, ACF-1 exhibits remarkable interconnectivity even after 5000 bending cycles, while ACF-2 experiences peeling after a mere 200 cycles. This suggests that ACF-1 boasts superior mechanical performance, particularly in terms of adhesion. In terms of electrical properties, ACF-1 maintains a negligible contact resistance even after 600 bending cycles (less than 1 Ω), but this resistance increases significantly after 800 cycles. This implies that ACF-1 can sustain exceptional and consistent electrical performance for a limited number of bending cycles, but as the number of cycles increases, the contact resistance rises, leading to electrical disconnection. On the other hand, ACF-2’s conductivity exhibits a tendency to decrease after 100 bending cycles, when the interconnect substrates remain intact. It indicates that ACF-2’s electrical performance is less stable in a flexible mechanical environment.

Regarding the insulation resistance in Figure 7d, the impact of bending cycles on the insulation resistance of ACF is negligible. This implies that conductive particles in the horizontal direction do not maintain continuous contact with conductors, even after a limited number of bending cycles.

### 3.4. Bending Simulation Results

**Figure 8:**
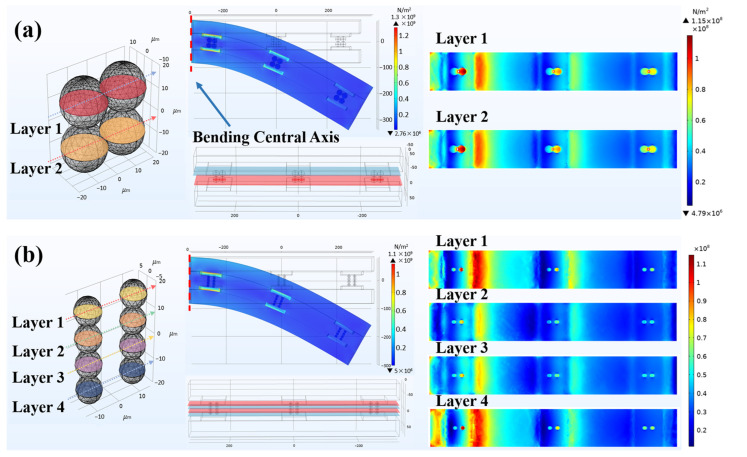
The results of bending test of (**a**) ACF-1 and (**b**) ACF-2, and the distribution of the von Mises force when subjected to a force of 0.2 N.

During the bending process, the most representative are the three groups of copper pads with conductive particles near the bending central axis. Figure 8a,b show the bending test results of the two ACFs and the distribution of the von Mises force when subjected to the same force of 0.2 N. It is noteworthy that the size differences of conductive particles do not affect the degree of bending of the FOF components. From the *Z*-axis perspective, the von Mises force distribution of ACF-1 in bending mechanics behavior remains consistent for conductive particles of varying layers. This suggests that the overall force is relatively uniform. while the von Mises force distribution of ACF-2 of different layers (four layers in total) differs, with a greater variance between layers on the top and bottom (layers 1 and 4) and layers in the middle (layers 2 and 3).

The principal stress in the main plane is the normal stress, which excludes the contribution of shear stress. The first principal stress, characterized by the highest value, is the most representative. In the simulation model, the separation of objects is primarily determined by the force components on the *Z*-axis. Therefore, this simulation investigates the Z-directional component of the first principal stress.

Theoretically, in the absence of any mechanical damage, electrical failure can occur in two main forms: separation of conductive particles from the copper electrodes or separation between the conductive particles themselves. Both of these forms cause discontinuity in the contact between the conductive particles and the copper electrodes. According to the simulation results, as the contact area of two conductive particles is under similar stress situations (as seen in Figure S4), there is no tendency for separation between the particles. On the other hand, the contact interface between the electrodes and the conductive particle is subjected to varying forces, which can cause separation. Consequently, the electrical failure during the bending process may primarily arise from the interface between conductive particles and the copper electrodes.

**Figure 9:**
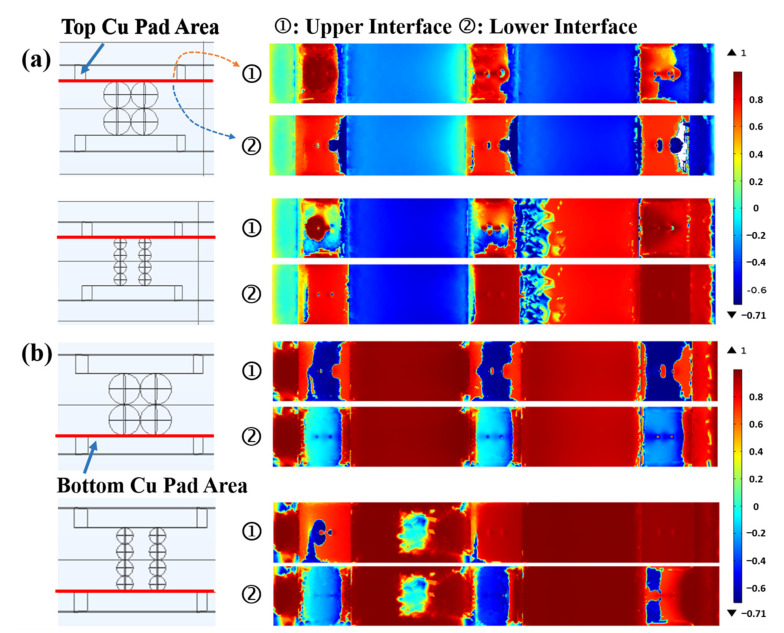
The first principal stress in the *z*-axis direction at (**a**) the contact interface between the top copper pad and the conductive particles and (**b**) the contact interface between the bottom copper pad and the conductive particles.

Using the contact interface between a conductive particle and a copper electrode as a reference, Figure 9a,b present the first principal stress distribution in the contact interface between copper electrodes and conductive particles after bending under a force of 0.2 N for the two ACFs. The red color in the figure denotes the positive *Z*-axis direction, while the blue color indicates the negative *Z*-axis direction. When the upper contact interface is red and the lower contact interface is blue, it suggests a tendency to separate during the bending process. For ACF-1, the stress distribution of the upper and lower contact interfaces in the top area is the same, indicating no separation trend. Meanwhile, both contact interfaces in the bottom Cu pad area have a tendency to squeeze each other, indicating a closer contact between the Cu pad and the conductive particle during bending. Therefore, there is no tendency to separate in ACF-1. For ACF-2, the stress distribution of both contact interfaces in the top Cu pad area is almost identical. However, the stress at the upper contact interface in the bottom Cu pad area is positive, indicating tensile stress applied to this interface, while the stress at the lower contact interface is negative, indicating pressure stress. This implies that there is a tendency to separate in the bottom Cu pad area of ACF-2. In summary, ACF-2, with smaller-sized conductive particles, theoretically has a higher likelihood of electrical failure.

### 3.5. The Failure Mechanism during Bending Process

Combining bending experiments with bending simulation results, the failure mechanism during the bending process can be summarized into two categories. Firstly, the cracking of the coating on conductive particles and the expansion of cracks lead to an increase in contact resistance. Secondly, the reduction in the contact area between conductive particles and electrodes results in an increase in contact resistance.

Bending cyclic testing can be understood as a periodic motion. Each bending cycle can be divided into two parts: bending and recovery. During the bending process, the FOF assembly is subjected to a bending moment along the direction of deformation. During the recovery process, the FOF assembly releases the bending moment in the direction of deformation recovery. This implies that conductive particles within the FOF assembly experience compression stress and tensile stress sequentially during a single bending cycle. When conductive particles are subjected to compression stress, micro-deformation occurs on the surface of the particles’ coating, leading to micro-cracks. Subsequently, when subjected to tensile stress, the micro-cracks in the coating are extended. As a result, conductive particles undergo repeated compression and tensile stress, leading to the generation and continuous expansion of new micro-cracks, ultimately merging into macro-cracks. These cracks formed on the coating will lead to an increase in contact resistance.

In addition to causing coating cracks, the bending cyclic process also changes the morphology of conductive particles, reducing the contact area between particles and electrodes. During the bonding process of FOF assemblies, conductive particles transform from spherical shapes to drum shapes under pressure. At this point, the shape of the contact part between the particles and the electrode is circular. After experiencing repeated cycles of compression and tensile stress, conductive particles change from drum shapes to irregular shapes, and the coating becomes fragmented. Consequently, the shape of the contact part between particles and electrodes becomes irregular, and the contact area decreases.

In the literature, many researchers have devoted their efforts to the development of novel ACFs to enhance the bending performance and fatigue reliability of ACFs [46,47]. Yoon et al. have been actively involved in the development of new ACFs and ACF packaging structures [15]. In recent years, a novel CIF (Chip-in-Flex) packaging scheme for APL (Anchoring Polymer Later) ACFs has been proposed [48,49]. It is reported that CIF assemblies using APL ACFs can undergo dynamic bending cycles of up to 150,000 with a bending radius of 6 mm, demonstrating excellent bending reliability. Although the maximum number of bending cycles (up to 5000) in this study is lower than that in the aforementioned research, considering that this study uses traditional single-layer ACFs and does not adopt CIF packaging, the FOF assembly using ACF-1 did not experience delamination or electrical failure after 5000 bending cycles. This result is encouraging for traditional single-layer ACFs, indicating that single-layer ACFs containing large-sized particles still possess good bending performance and fatigue reliability.

On the other hand, Chao-Ming Lin et al. have studied the bending behavior and resistance characteristics of Flex (PI with Cu)-On-Film (ITO-coated PET) packaging, considering the flexible effect under static bending loads during continuous bending cycles [28]. Although these findings regarding ACF bending tests are inspiring, progressive, and intriguing, the influence of ACF conductive particle size on the bending performance of FOF components remains unknown and worthy of investigation. The following part will elucidate the mechanism of failure due to the size effects of the ACF conductive particle during the bending cycle process.

Smaller-sized conductive particles may accelerate the occurrence of electrical failure. Firstly, due to the greater curvature of smaller-sized particles, the Au/Ni coating of smaller-sized particles is more likely to crack during the same bending cyclic process. This situation leads to a rapid increase in the resistance of FOF assemblies using ACF-2 after very few bending cycles, as shown in Figure S3. Secondly, Figure 9 shows the distribution of the first principal stress of the contact interface during a single bending process (excluding the recovery process). As described in Section 3.4, there is already a trend of separation between the Cu pad and ACF after a single bending cycle for FOF assemblies using smaller-sized particles. This indicates that after multiple bending cycles, the contact area between conductive particles in ACF and electrodes will rapidly decrease, and debonding may occur. In fact, FOF assemblies using ACF-2 demonstrated the above failure theory in bending tests. FOF components using smaller-sized particles experienced debonding after 200 bending cycles.

## 4. Conclusions

In this study, the mechanism of the size effects of the ACF conductive particles under flexible mechanical conditions was investigated. First, the feasibility of employing two commercially accessible ACFs as interconnection materials for flex-on-flex (FOF) assemblies was studied via a low-pressure thermo-compression bonding technique. An in-house-made rapid electrical test platform was applied to evaluate the electrical performance of nine FOF assemblies with different bonding parameters. Our findings reveal that the low-pressure bonding process is compatible with both tested ACFs. With a 0.67 MPa bonding force, the contact resistances of the interconnections are less than 1 Ω, even after 600-cycle mechanical stressing. Furthermore, we found the optimal bonding parameters that achieve the best electrical performance. These bonding parameters are: 170 °C and 0.67 MPa for ACF-1, versus 220 °C and 0.67 MPa for ACF-2.

Second, combining experimental and computational studies of ACF’s bending properties, we concluded two failure modes leading to the FOF assemblies during the bending. First, cracks form in the coating layer of the conductive particles, and the expansion of these cracks leads to an increase in contact resistance. Second, the contact area reduction between the conductive particles and electrodes results in an increase in contact resistance.

More importantly, we discovered that smaller conductive particles would accelerate the occurrence of electrical failure because, first, the coatings of these smaller particles are more likely to crack during the same bending process due to the greater curvature. Second, separation between the Cu pad and ACF (with smaller filler particles) showed up after the first bending cycle, based on the bending simulation results, which indicates that FOF assemblies using smaller particles are more susceptible to resistance increase and even electrical faults in the early stages of bending cyclic testing.

These findings shed light on the impact of conductive particle size on ACF performance under flexible mechanical environments and may facilitate the development of more reliable electronic packaging materials.

## Figures and Tables

**Table 1:** The standard bonding parameters of the two ACFs.

ACF Type	Temporary Bonding			Final Bonding		
	Pressure (MPa)	Temperature (°C)	Time (s)	Pressure (MPa)	Temperature (°C)	Time (s)
ACF-1	1	80	2	0.67	150	15
ACF-2	1	80	2	0.67	200	10

**Table 2:** The modified bonding parameters of the two ACFs.

ACF Type	Temporary Bonding			Final Bonding		
	Pressure (MPa)	Temperature (°C)	Time (s)	Pressure (MPa)	Temperature (°C)	Time (s)
ACF-1	Same as the standard bonding parameters			0.20	130, 150, 170	15
ACF-1				0.33	130, 150, 170	15
ACF-1				0.67	130, 150, 170	15
ACF-2	Same as the standard bonding parameters			0.20	180, 200, 220	10
ACF-2				0.33	180, 200, 220	10
ACF-2				0.67	180, 200, 220	10

**Table 3:** Properties of the materials used in the simulation.

Part	Material	Young’s Modulus (GPa)	Density (kg·m^−3^)	Poisson’s Ratio	Thickness (μm)
* Flex. Sub.	PI (Polyimide)	2.5	1500	0.34	50
Electrode	Cu	110.0	8960	0.35	1.2
Resin matrix	Epoxy resin	2.0	2000	0.4	40
Conductive particles	PP (Polypropylene)	8.9	900	0.4203	20 (diameter) 10 (diameter)

* The term “Flex. Sub.” refers to the Flexible Substrate, which is not employed in bonding simulations but exclusively utilized in bending simulations.

## Data Availability

Data are contained within the article and Supplementary Materials.

## Supplementary Materials

The following supporting information can be downloaded at: https://www.mdpi.com/article/10.3390/ma17071658/s1, Figure S1: Results in the electrical properties on temperature variable group (TVG) investigation (PDF); Figure S2: Results in the electrical properties on pressure variable group (PVG) investigation (PDF); Figure S3: Contact resistance of ACF-2 varies with the increasing number of bending cycles (PDF); Figure S4: The stress distribution in the contact area of conductive particles at the *Z*-axis direction (PDF). Video S1: A video of the procedure of the bending test experiment (MP4).
