# Supplementary material for: Size Effects of Au/Ni-Coated Polymer Particles on the Electrical Performance of Anisotropic Conductive Adhesive Films under Flexible Mechanical Conditions
Source: Materials (Basel). 2024 Apr 4;17(7):1658. doi: 10.3390/ma17071658 (PMC11012521; doi:10.3390/ma17071658)
Supplement: Supplementary file 1 [file materials-17-01658-s001.zip › Figure S1-S4 and Screening Criteria.pdf]

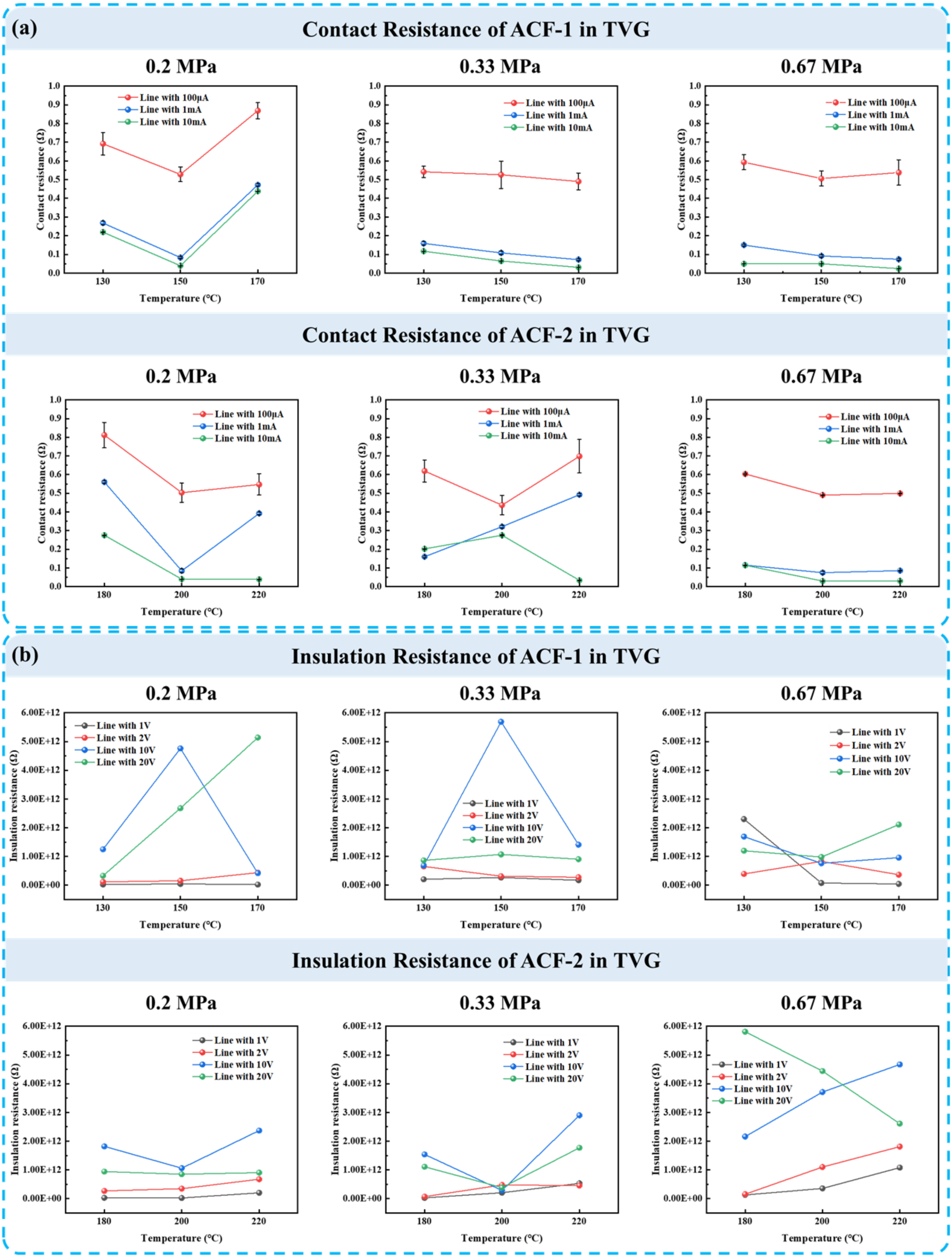


**Figure S1.** Results in the electrical properties on temperature variable group (TVG) investigation. (a) the contact resistance of ACF-1 and ACF-2 and (b) the insulation resistance of ACF-1 and ACF-2 with increasing bonding temperature (ACF-1: 130 ℃, 150 ℃ and 170 ℃; ACF-2: 180 ℃, 200 ℃ and 220 ℃).

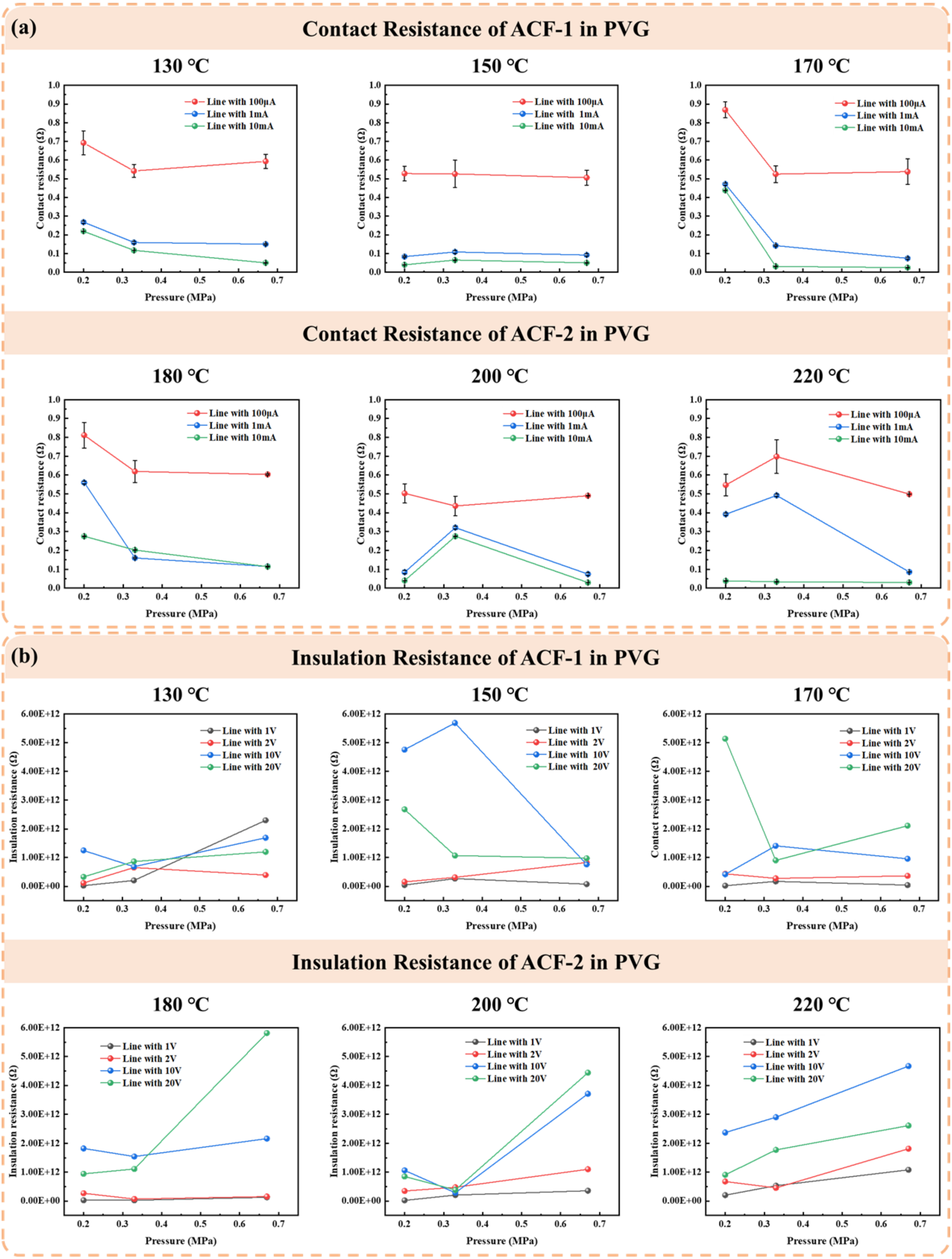


**Figure S2.** Results in the electrical properties on pressure variable group (PVG) investigation. (a) the contact resistance of ACF-1 and ACF-2 and (b) the insulation resistance of ACF-1 and ACF-2 with increasing bonding pressure: 0.20 MPa, 0.33 MPa and 0.67 MPa.

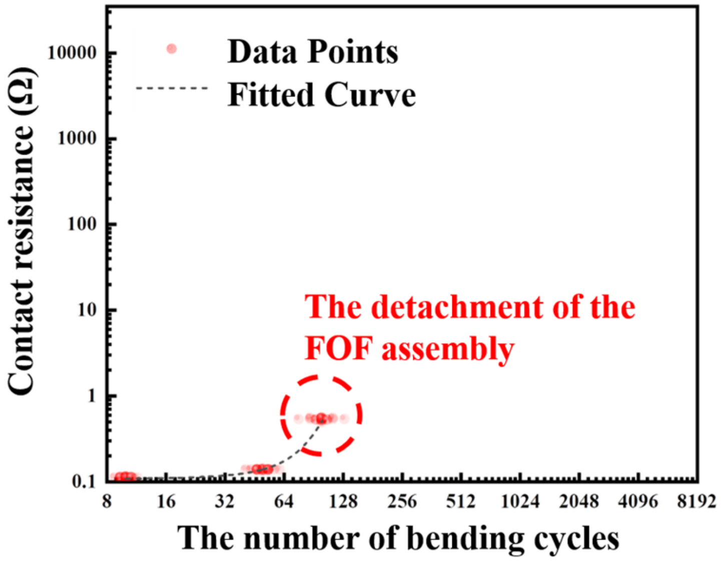


**Figure S3.** Contact resistance of ACF-2 varies with the increasing number of bending cycles.

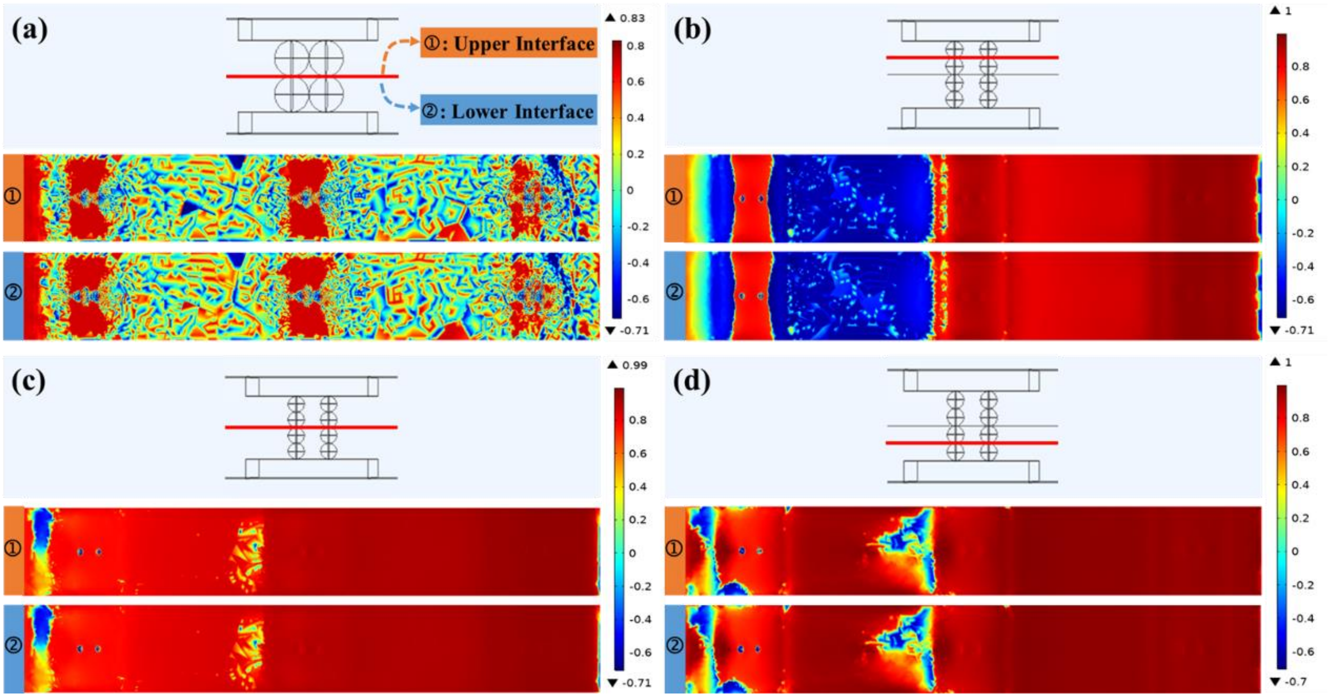


**Figure S4.** The stress distribution in the contact area of conductive particles at the Z-axis direction. (a) the strain distribution in ACF-1; (b) (c) (d) the strain distribution in ACF-2. As the contact area of the two conductive particles for every layer is under similar stress situations, there is no tendency for separation between the particles according to theory.

# Screening Criteria

As depicted in Figure S1 or S2, when considering all other bonding parameters as constant, it has been observed that the contact resistance and resistance data fluctuation (as indicated by the size of the error bars) of a ACF decrease as the applied current value increases. This intriguing phenomenon can be attributed to the limitations of the digital source meter employed in this study. Specifically, when a low magnitude of current is applied, the resulting voltage measurement is correspondingly small due to the constant resistance. As the voltage becomes as low as the measurement accuracy of the instrument, a substantial data error becomes apparent. Therefore, the contact resistance values obtained at applied current levels of 10mA in this experiment are closer to the true values.

Under the same bonding parameters, the insulation resistance of the same ACF increases as the applied voltage increases. This phenomenon can be attributed to the large insulation resistance value (on the order of $10^{12}$ Ω). When a small voltage is applied to measure the current, the resulting current value is very small, reaching the picoampere (pA) level. Consequently, the current in the circuit may be lower than the current measurement accuracy of the digital source meter, leading to a measured resistance value smaller than the actual value. Moreover, considering the small size of the test substrate, it is crucial to prevent excessive electric field and potential breakdown of the sample. Therefore, the applied voltage should not be set too high.

In this experiment, the digital source meter with a current measurement accuracy of 100 pA demonstrates excellent accuracy even when measuring current in the nanoampere (nA) range. Consequently, the values obtained at applied voltage levels of 20 V in this experiment are closer to the true values.
